# *N*^4^-Methylcytosine Supports the Growth of *Escherichia coli* Uracil Auxotrophs

**DOI:** 10.3390/ijms26051812

**Published:** 2025-02-20

**Authors:** Jaunius Urbonavičius, Aušrinė Čekytė, Daiva Tauraitė

**Affiliations:** Department of Chemistry and Bioengineering, Vilnius Gediminas Technical University, Saulėtekio Av. 11, 10223 Vilnius, Lithuania; jaunius.urbonavicius@vilniustech.lt (J.U.); ausrinecekyte@gmail.com (A.Č.)

**Keywords:** *Escherichia coli*, modified heterocyclic base, uracil auxotrophy, methylcytosine, metabolism

## Abstract

*N*^4^-methylcytosine is a modified heterocyclic base present both in RNA and DNA. The biosynthesis and function of this derivative are widely investigated. However, how the demethylation of this base occurs is not known. Here, we have investigated the growth of an *Escherichia coli* uracil auxotroph strain in minimal M9 medium supplemented with *N*^4^-methylcytosine. We have found that this compound, but not the related *N*^4^,*N*^4^-dimethylcytosine, well supports growth with a generation time of the bacterium being 3 h compared to 1.5 h for media supplemented with cytosine or uracil. Using high-performance liquid chromatography (HPLC), we have demonstrated that the concentration of *N*^4^-methylcytosine in the growth medium decreases by 12% after 24 h of growth. We have shown that *N*^4^-methylcytosine is not directly converted into uracil by *E. coli* CodA cytosine deaminase. Instead, we propose the enzymatic pathway in which *N*^4^-methylcytosine is converted into cytosine by yet unknown demethylase, whereas CodA converts the resulting cytosine to uracil, thereby supporting the growth.

## 1. Introduction

RNA is a molecule involved in many cellular functions. To fulfill its function, different RNA species are modified by various chemical groups. The biggest variety of modifications is observed for tRNA, where more than 170 different species are found [1]. These modifications play an important role in the maintenance of tRNA structure and stability and for their localization and transport within the cell. The links between tRNA modifications, cellular stress response, protein synthesis, and disease development have also been extensively investigated, and some modifications are being used in new types of mRNA vaccines [2,3]. Despite numerous previous works on the discovery of the genes that encode the enzymes that catalyze the formation of RNA modifications and on their role in the cell, much less is known about the degradation and return to metabolism of modified nucleosides and the corresponding heterocyclic bases. Studying such enzymes is of great importance as they can be used as biocatalysts in e.g., the biosynthesis of antiviral drugs [4]. Demodification of the heterocyclic bases is often investigated at the level of RNA or DNA as part of epigenetics studies [5]. However, degradation of the corresponding modified nucleosides was investigated only in some cases. One example is the conversion of pseudouridine into uridine and then into uracil [6]. In the case of modified heterocyclic bases, the best-studied compound is 5-methyluracil (thymine), which is known to be degraded in three distinct pathways (Rut, oxidative, and reductive ones [7,8,9].

Despite the presence of the majority of modified nucleosides in tRNA, some of the modifications are found in other RNA types or in DNA. One such modification is *N*^4^-methylcytidine (m^4^C), found at position 1402 of 16S rRNA in the bacterium *Escherichia coli.* It may also be further 2′-O-methylated to form m^4^Cm [10]. These authors demonstrate that loss of the *N*^4^-methyl group of m^4^Cm1402 in the *rsmH* mutant that is lacking the corresponding methyltransferase leads to a 15% increase in doubling time compared with the wild-type strain. In this mutant, the efficiency of non-AUG initiation and the fidelity of translation are also affected. For the plants, it was found that in *Arabidopsis* chloroplasts, m^4^C is present at position 1352 of 16S rRNA. Loss of CMAL protein, responsible for the introduction of such modification, results not only in impaired function of chloroplasts but also affects leaf and root development [11]. It was also demonstrated that METTL15 methyltransferase is responsible for the formation of m^4^C at position 839 of mitochondrial 12S rRNA in human cells, and its lack impairs the translation of human mitochondria. In addition, m^4^_2_C was found in the total RNA of Zika and HCV virions during the infection of human cells [12]. Recently, m^4^_2_C was observed to be present at position 918 in the 16S rRNA of the archaeon *Thermococcus kodakarensis* [13]. In many bacteria, m^4^C is found as one of three (together with m^5^C and m^6^A) DNA epigenetic modifications [14]. In *Helicobacter pylori*, m^4^C DNA methylation regulates transcription and pathogenesis [15]. Recently, it was found that such DNA modification can be used as an epigenetic mark in eukaryotes, namely bdelloid rotifers, tiny freshwater invertebrates [16].

To investigate the degradation of modified nucleotides into corresponding heterocyclic bases and further demodification and return into the metabolism, we used an *E. coli* uracil auxotrophy-based selection system where the inability of modified heterocyclic bases to support the bacterial growth was reversed by the enzymatic activity of proteins that convert them into uracil. Using such an approach, a gene that encodes the TudS protein catalyzing the conversion of 2-thiouracil into uracil was found [17]. Our systematic screening of several naturally occurring and synthetic modified heterocyclic bases for support of the growth of *E. coli* uracil auxotrophs demonstrated that *N*^4^-methylcytosine, but not *N*^4^,*N*^4^-dimethylcytosine, performs this role. Below we describe the experiments that confirm our observations and propose a mechanism for the conversion of *N*^4^-methylcytosine into uracil.

## 2. Results and Discussion

### 2.1. N^4^-Methylhcytosine but Not N^4^,N^4^-Dimethylcytosine Supports Growth of the Uracil Auxotrophs

#### 2.1.1. Growth of Uracil Auxotrophs in M9 Solid Medium

**Figure 1:**
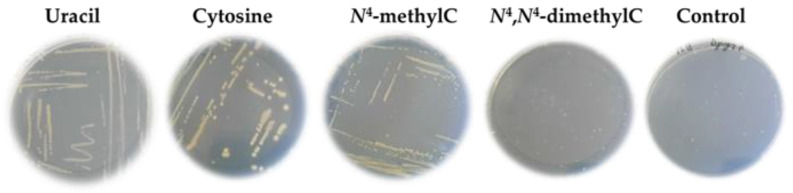
Growth of the *E. coli* ∆*pyrF*::Km strain on the M9 agar minimal medium supplemented with either uracil, cytosine, *N*^4^-methylcytosine, *N*^4^,*N*^4^-dimethylcytosine, or without any heterocyclic base (control). The agar plates were incubated at 37 °C for 72 h.

As the initial experiments, we have investigated several naturally occurring and synthetic pyrimidine heterocyclic bases for their role in supporting the growth of uracil auxotrophs. This assay is based on the assumption that some of the tested compounds are converted by cellular enzymes into uracil from the modified uracils directly or, as in the case of modified cytosines, via cytosine by aid of the bacterial cytosine deaminases. We found that *N*^4^-methylcytosine well supported the growth of the *E. coli* ∆*pyrF*::Km auxotroph strain compared to the other compounds tested. However, the dimethylated derivative *N*^4^,*N*^4^-dimethylcytosine did not support its growth (Figure 1). Similar growth patterns were observed for the *S. typhi* ∆*pyrF*::Tn10 uracil auxotroph, suggesting that this organism uses the same strategy for conversion of *N*^4^-methylcytosine into uracil (Figure S1). However, no growth was observed in the case of the *B. subtilis* ∆*pyrF*::erm uracil auxotroph (Figure S2).

#### 2.1.2. Growth of the *E. coli* ∆*pyrF*::Km in M9 Agar Medium After the Serial Dilutions

**Figure 2:**
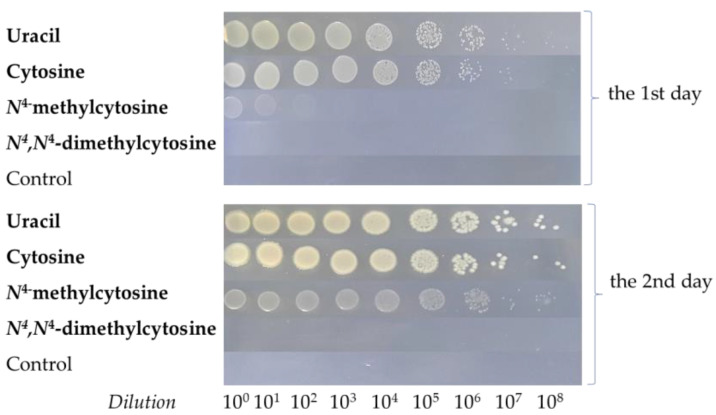
Growth of the *E. coli* ∆*pyrF*::Km strain in the M9 agar medium supplemented with either uracil, cytosine, *N*^4^-methylcytosine, or *N*^4^,*N*^4^-dimethylcytosine after serial dilutions.

For better comparison of growth rates of the *E. coli* ∆*pyrF*::Km strain in M9 solid medium supplemented with the tested above-modified cytosines, serial dilutions of cells obtained from the overnight culture were plated on the agarized M9 medium with the same modified bases and controls as described above. Figure 2 demonstrates that after 1 day (approx. 24 h) of growth, a similar growth rate was observed when either uracil or cytosine was used as substrates, whereas only some growth was observed for *N*^4^-methylcytosine. At the same time, no growth was observed when *N*^4^,*N*^4^-dimethylcytosine was used as a source of uracil or any heterocyclic base was not added. However, after 2 days (approx. 48 h), clearly visible growth in the medium with *N*^4^-methylcytosine was observed, whereas both uracil and cytosine strongly supported the growth. As after 1 day, no growth was observed when *N*^4^,*N*^4^-dimethylcytosine or no heterocyclic base was added.

#### 2.1.3. Determination of the *E. coli* ∆*pyrF*::Km Generation Time

**Figure 3:**
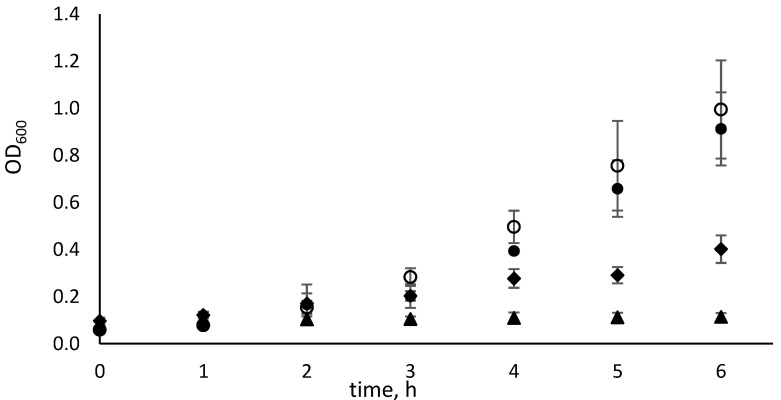
Growth of the *E. coli* ∆*pyrF*::Km strain in the M9 liquid medium supplemented with uracil 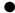; cytosine 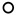; *N*^4^-methylcytosine 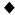; or *N*^4^,*N*^4^-dimethylcytosine 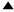. Error bars show the standard deviation of the three experiments.

To determine the generation time, bacteria were grown overnight in M9 liquid medium with uracil. After harvesting and washing the cells in 0.9% NaCl, they were transferred into fresh medium supplemented with respective substrates. Afterwards, growth in each medium was spectrophotometrically measured to obtain the respective growth curves. Figure 3 demonstrates good growth of the tested strain in media containing either uracil or cytosine and somewhat slower growth for *N*^4^-methylcytosine. By contrast, very little, if any, growth was observed when *N*^4^,*N*^4^-dimethylcytosine was used as a substrate. For better comparison of the growth rates in different media, generation times were calculated and are presented in Table 1. The semi-logarithmic plots used for the calculation of generation times are presented in Figure S3. Our results indicate that in the presence of uracil or cytosine, the generation time of the *E. coli* ∆*pyrF*::Km strain in M9 medium is around 1.5 h, whereas in the presence of *N*^4^-methylcytosine, the generation time is around 3 h. However, when *N*^4^,*N*^4^-dimethylcytosine was used, a generation time of at least 33 h was obtained, suggesting that very little, if any, of this compound is consumed.

**Figure 4:**
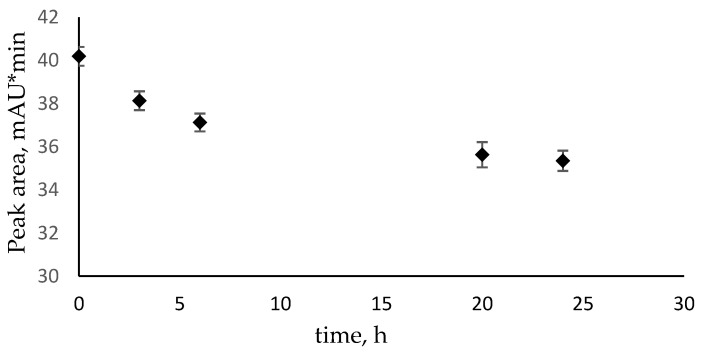
Decrease in *N*^4^-methylcytosine concentration during the growth of the *E. coli* ∆*pyrF*::Km strain in M9 liquid medium. The results are represented as peak area obtained during HPLC analysis. Error bars show the standard deviation of the three experiments.

To demonstrate that *N*^4^-methylcytosine is consumed during the growth of the *E. coli* ∆*pyrF*::Km strain in M9, samples of the medium obtained during the growth of bacteria were collected and analyzed by HPLC. Figure 4 demonstrates that the concentration of *N*^4^-methylcytosine rapidly decreases during the first six hours of growth and continues to decrease during the rest of the observed growth period. HPLC chromatograms representing the decrease in the peak area are shown in Figure S4. After 24 h of growth, around 12% of *N*^4^-methylcytosine was consumed and presumably converted into cytosine, further into uracil, for supporting the growth.

One reason why the concentration of *N*^4^-methylcytosine decreases in the growth medium during the growth of bacteria could be the instability of this compound at the experimental conditions used. To test this, *N*^4^-methylcytosine or *N*^4^,*N*^4^-dimethylcytosine-containing medium was incubated at the same growth conditions without bacteria for 72 h, and concentrations of *N*^4^-methylcytosine, *N*^4^,*N*^4^-dimethylcytosine, cytosine, and uracil were measured by HPLC. Figure S5 demonstrates no degradation of *N*^4^-methylcytosine or *N*^4^,*N*^4^-dimethylcytosine and the appearance of additional cytosine and uracil peaks in the HPLC profile, attesting that *E. coli* cells are responsible for the decrease in the concentration of the heterocyclic base under investigation.

**Figure 5:**
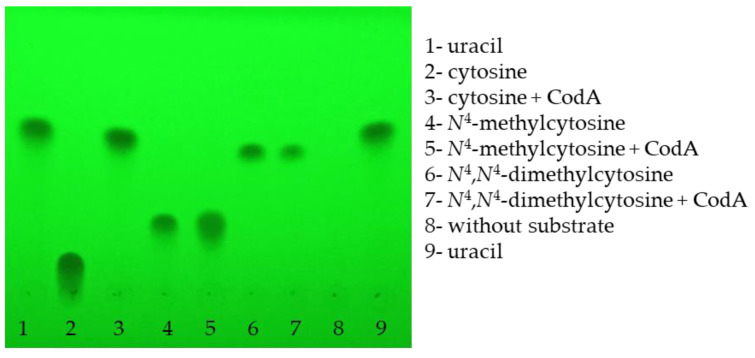
Cytosine deaminase CodA does not convert *N*^4^-methylcytosine or *N*^4^,*N*^4^-dimethylcytosine into uracil. Thin-layer chromatography (TLC) plate of cytosine, *N*^4^-methylcytosine, or *N*^4^,*N*^4^-dimethylcytosine enzymatic reactions with CodA.

**Figure 6:**
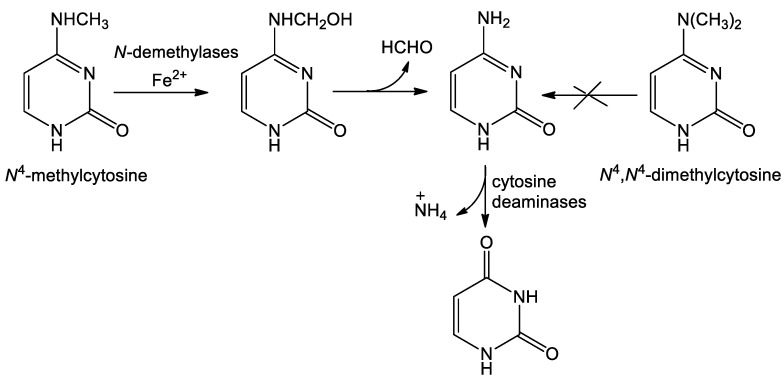
Proposed mechanism of *N*^4^-methylcytosine conversion into uracil.

When looking for the enzymes capable of catalyzing the conversion of *N*^4^-methylcytosine into uracil in *E. coli* and thereby supporting the growth in M9 minimal medium, one possibility would be direct conversion without the formation of a cytosine intermediate. The candidate to perform this reaction is a well-characterized CodA deaminase that catalyzes the conversion of cytosine into uracil [18]. To test whether CodA catalyzes the conversion of *N*^4^-methylcytosine into uracil, the enzyme was purified, and its enzymatic activity was tested using the same substrates as the ones used for the growth rate determination. Figure 5 demonstrates that whereas cytosine was well converted into uracil, no such conversion was observed for *N*^4^-methylcytosine (and also for *N*^4^,*N*^4^-dimethylcytosine), suggesting that *E. coli* encodes the specialized enzyme to convert *N*^4^-methylcytosine into cytosine, and only then the latter is converted into uracil by CodA (Figure 6). This kind of mechanism may be conserved at least in bacteria, since similar to the *E. coli* ∆*pyrF*::Km strain, growth patterns were observed for the *S. typhi* ∆*pyrF*::Tn10 uracil auxotroph. Also, it is possible that in *B. subtilis*, *N*^4^-methylcytosine is also converted into cytosine, but because of the absence of cytosine deaminase in the strain tested, it cannot support the growth in minimal media requiring uracil. Indeed, we have demonstrated that in contrast to *E. coli* and *S. typhi*, cytosine does not support the growth of the *B. subtilis* ∆*pyrF*::erm uracil auxotroph (Figure S2).

How *N*^4^-methylcytosine is converted into cytosine is unclear, even at the level of nucleosides, nucleotides, or nucleic acids. It was previously demonstrated that some cytidine deaminases are capable of converting both *N*^4^-methylcytidine and *N*^4^,*N*^4^-dimethylcytidine into uridine [19]. However, it is likely that *N*^4^-methylcytidine is not a physiological substrate of cytidine deaminase isolated from *E. coli* B, since the observed K_m_/V_max_ values were at least three orders of magnitude higher than those of cytidine deamination [20]. For a long time, the m^4^C modification was considered as being part of the bacterial restriction/modification system [21]. However, the presence of this modification in transposable elements of the freshwater invertebrate *Adineta vaga* was associated with their transcriptional repression [16]. In that work, N4CMT, a horizontally transferred enzyme of bacterial origin, was identified as DNA m^4^C methyltransferase (“writer” enzyme). However, the demethylation (“eraser”) enzyme for this modification is unknown. Recently, it was suggested [22] that its catalytic activity may be similar to that of AlkB proteins that are responsible for m^6^A demethylation in RNA. Indeed, both AlkB family non-heme Fe(II)/2-oxoglutarate-dependent oxygenases FTO and ALKBH5 act as oxidative m^6^A demethylases, but their action yields different products, hm^6^A and A, respectively [23]. In the case of small molecules, N-dealkylation is often performed by the cytochrome P450 superfamily of heme-containing monooxygenases [24]. Bacterial P450 was successfully used to convert drug verapamil into norverapamil [25]. Therefore, one may envision the following scenario of *N*^4^-methylcytosine demethylation to yield the cytosine and then the uracil (Figure 6). As mentioned above [19], *N*^4^,*N*^4^-dimethylcytidine may be a substrate for conversion into uridine by cytidine deaminases. Here, we propose different reaction mechanisms for the removal of *N*^4^-methyl groups. At least two *N*-dimethylated heterocyclic bases (m^6^_2_A and m^2^_2_G, see [1]) exist in RNA. It was demonstrated that ALKBH5 catalyzes demethylation of m^6^_2_A using rRNA or oligoribonucleotides as substrates [26]. However, removal of the first methyl group may be the rate-limiting step (see Figure 4D in [27]). Demethylation of *N*^4^,*N*^4^-dimethylcytosine may occur in a similar manner, which is reflected in the slow, if any, conversion into *N*^4^-methylcytosine and further into cytosine and uracil to support the growth of uracil auxotrophs.

*E. coli* is one of the most thoroughly characterized model organisms, yet around 35% of genes in its genome [28] are poorly described. In EcoCyc, the most comprehensive curated genome database for *E. coli* K-12 [29], 15.5% (738) of genes are annotated as the uncharacterized ones. Therefore, several approaches may be used in search of the *E. coli N*^4^-methylcytosine demethylase gene. One way would be the use of the gene ontology (GO) resource [30] to search for the candidate genes and then test the enzymatic activity of the corresponding proteins. Another way is the use of traditional protein chromatography following the proposed enzymatic activity [31]. However, this method may be tedious since no recombinant proteins are used for the initial screening procedures. The third way is genome-wide screening for the mutants that are not able to grow in *N*^4^-methylcytosine-containing minimal media using a single gene deletion library, similar to what was previously used [32]. This approach may also be time-consuming; however, budding yeast are particularly useful since the genetic background of the traditionally used *Saccharomyces cerevisiae* BY4741 strain renders it the uracil auxotroph. For bacteria, a more plausible approach is to use transposon insertion sequencing, a currently well-established method [33]. Finally, the transcriptomics approach similar to what was used to discover and characterize the morphinan N-demethylase [34] may be suggested.

## 3. Materials and Methods

### 3.1. Synthesis of Methylated Cytosines

General information

Chemicals, reagents, solvents, and kits were purchased from Sigma-Aldrich (Merck group, Darmstadt, Germany) and Thermo Fisher Scientific Baltics (Vilnius, Lithuania) and used without further purification. *N*^4^-methylcytosine and *N*^4^,*N*^4^-dimethylcytosine were synthesized and analyzed as described below. Thin-layer chromatography (TLC) was carried out on 25 TLC aluminum sheets coated with silica gel 60 F_254_ (Merck, Darmstadt, Germany), and column chromatography was performed on silica gel 60 (0.063–0.200 nm) (Merck) using chloroform/methanol mixtures as a mobile phase. NMR spectra were recorded in DMSO-d_6_ on a Bruker Ascend 400 ^1^H NMR—400 MHz and ^13^C NMR—101 MHz (Bruker BioSpin, Rheinstetten, Germany). Chemical shifts were reported in ppm relative to the solvent resonance signal as an internal standard. UV spectra were recorded on a SPECORD 210 UV/Vis spectrophotometer (analytic Jena, Jena, Germany). The HPLC analyses were performed using a high-performance liquid chromatography system (UFLC LC-20AD), equipped with a photodiode array detector (SPD-M20A, Shimadzu, Kyoto, Japan). The chromatographic separation was conducted using a Symmetry C18 column, 3.9 × 150 mm (Waters, Milford, MA, USA) at 40 °C and a mobile phase that consisted of a 0.01% formic acid (or 5 mM ammonium acetate) and water solution (solvent A) and a 0.01% formic acid (or 5 mM ammonium acetate) and methanol solution (solvent B).

#### 3.1.1. Synthesis of N^4^-Methylcytosine

(i) 4-methylthiouracil

To a solution of 120 mg (0.94 mmol) 4-thiouracil and 0.94 mL 1 M NaOH in 5.0 mL 50% ethanol, 116 μL (1.88 mmol) methyl iodide was added, and the mixture was stirred at room temperature for 1 h. After the reaction was completed (TLC), the mixture was neutralized with diluted acetic acid (25% in water) to approx. pH 6. After evaporation under reduced pressure, the residue was purified by flash column chromatography (silica gel, chloroform/methanol mixture, 10/0→10/1). Yield 108 mg (81%), yellowish foam, R_f_ = 0.46 (CHCl_3_/MeOH–9/1). UV (CH_3_OH) λ_max_ (ε) 297 (11.2 × 10^3^) nm (M^−1^ × cm^−1^). ^1^H NMR (DMSO-d6, 400 MHz): δ = 2.43 (s, 3H, CH_3_), 6.32 (d, 1H, *J* = 6.7 Hz, CH=CH), 7.62 (d, 1H, *J* = 6.7, Hz, CH=CH), 11.47 (s, 1H, NH). ^13^C NMR (DMSO-d6, 101 MHz): δ = 12.39, 102.22, 143.60, 154.71, 177.93.

(ii) *N*^4^-methylcytosine

To a solution of 50 mg (0.35 mmol) 4-methylthiouracil in 5 mL of water, 542 μL (7 mmol) of 40% CH_3_NH_2_ in water was added, and the mixture was stirred at 70 °C for 36 h. After the reaction was completed (TLC), the solvents were removed under reduced pressure. The residue was purified by flash column chromatography (silica gel, chloroform/methanol mixture, 10/0→10/2). Yield 39 mg (90%), white solid, R_f_ = 0.15 (CHCl_3_/MeOH–9/1). UV (CH_3_OH) λ_max_ (ε) 267 (12.1 × 10^3^) nm (M^−1^ × cm^−1^). ^1^H NMR (DMSO-d6, 400 MHz): δ = 2.73 (d, *J* = 4.5 Hz, 3H, CH_3_), 5.58 (d, 1H, *J* = 7.1 Hz, CH=CH), 7.24 (d, 1H, *J* = 7.1, Hz, CH=CH), 7.54 (d, 1H, *J* = 4.8 Hz, NH), 10.24 (s, 1H, NH). ^13^C NMR (DMSO-d6, 101 MHz): δ = 27.16, 93.71, 141.61, 157.26, 165.45.

#### 3.1.2. Synthesis of *N*^4^,*N*^4^-Dimethylcytosine

To a solution of 50 mg (0.35 mmol) 4-methylthiouracil in 5 mL of water, 805 μL (7 mmol) of 40% (CH_3_)_2_NH in water was added, and the mixture was stirred at 70 °C for 36 h. After the reaction was completed (TLC), the solvents were removed under reduced pressure. The residue was purified by column chromatography (silica gel, chloroform/methanol mixture, 10/0→10/2). Yield 46 mg (95%), white solid. R_f_ = 0.24 (CHCl_3_/MeOH–9/1). UV (CH_3_OH) λ_max_ (ε) 275 (13.4 × 10^3^) nm (M^−1^ × cm^−1^). ^1^H NMR (DMSO-d6, 400 MHz): δ = 3.02 (s, 6H, CH_3_), 5.88 (d, 1H, *J* = 7.4 Hz, CH=CH), 7.42 (d, 1H, *J* = 7.4, Hz, CH=CH), 10.44 (s, 1H, NH). ^13^C NMR (DMSO-d6, 101 MHz): δ = 90.38, 143.19, 156.38, 164.77.

### 3.2. Bacterial Strains and Growth Media

The uracil auxotroph ∆*pyrF*::Km in *Escherichia coli* K-12 BW25113 genetic background was obtained from the Keio collection via Horizon Discovery (Cambridge, UK). *Salmonella typhi* LT2 ∆*pyrF*::*Tn10* was a kind gift from G.R. Björk laboratory (Umeå, Sweden). The *Bacillus subtilis* 168 ∆*pyrF*::*erm trpC2* strain was obtained from the Bacillus Genetic Stock Center (Columbus, OH, USA).

Bacterial strains were recovered from long-term storage at −80 °C by plating them on LB-agar plates at 37 °C. For testing the growth of bacteria in minimal media, M9 medium, containing necessary salts and 0.4% of glucose, was supplemented with 20 µg/mL (or 200 µg/mL for the determination of concentration by HPLC) of either uracil, cytosine, *N*^4^-methylcytosine, or *N*^4^,*N*^4^-dimethylcytosine. In the case of *E. coli*, the growth medium was also supplemented with 15 µg/mL of kanamycin. For *B. subtilis*, media were supplemented with 40 µg/mL tryptophan since the *trpC2* mutation in the *B. subtilis* 168 strain renders cells the tryptophan auxotroph. For growing bacteria on solid media, 2% agar was added.

### 3.3. Growth of Bacteria in Minimal Media

For monitoring the bacterial growth in solid media, bacterial strains were inoculated by streaking them on M9 minimal media containing different modified cytosines and respective controls and aerobically grown for up to 7 days at 37 °C.

For better growth rate comparison in different media, the *E*. *coli* ∆*pyrF*::Km strain was grown in liquid M9 minimal medium supplemented with uracil and kanamycin overnight, OD_600_ was measured, the concentration of cells was adjusted to approximately 10^9^/mL (assuming that OD_600_ = 1 corresponds to 8 × 10^8^/mL) by washing and diluting in 0.9% NaCl, and serial 10× dilutions were made before plating 5 µL of cell suspension on M9 minimal medium agar plates supplemented with kanamycin, respective modified cytosines, and controls.

### 3.4. Determination of Growth Rate of E. coli ∆pyrF::Km Strain

The strain was grown in M9 nutrient minimal medium supplemented with uracil and kanamycin at 37 °C overnight. Cells were washed with a 0.9% NaCl solution and transferred to the same medium supplemented with kanamycin and 200 µg/mL of either uracil, cytosine, *N*^4^-methylcytosine, or *N*^4^,*N*^4^-dimethylcytosine, and cells were aerobically grown at 37 °C. Samples for OD_600_ measurements were taken each hour. Experiments were performed 3 times in triplicates. Generation times in different media were calculated by plotting the values obtained at the exponential growth conditions in a semi-logarithmic scale (Figure S3). From the obtained curves, the generation time g was calculated according to the formula

g = t × ln2/(lnXt − lnX_0_),

where t is the time in hours, Xt is OD_600_ at time t, and X_0_ is OD_600_ at time 0.

### 3.5. Determination of N^4^-Methylcytosine and N^4^,N^4^-Dimethylcytosine Concentration and Chemical Stability Using HPLC

Samples for HPLC analysis were taken under the same frequency as for the measurement of OD_600_. A total of 500 µL of sample was centrifuged, and 0.6 mL of methanol was added to 0.3 mL of the obtained supernatant in order to kill the remaining cells and to precipitate the salts. Samples were centrifuged again, and 10 μL of the solution was loaded on a C18, 3.9 × 150 mm column. HPLC analysis was performed as described above in Section 3.1.

For chemical stability analysis, 200 µg/mL *N*^4^-methylcytosine or *N*^4^,*N*^4^-dimethylcytosine was incubated in the M9 liquid medium supplemented with kanamycin at 37 °C for 72 h without the *E. coli* ∆*pyrF*::Km strain. Samples were taken each day (approx. every 24 h) and analyzed by HPLC.

### 3.6. Purification of E. coli CodA Protein and Determination of Its Enzymatic Activity

Recombinant CodA protein was purified using the pQE-70-*codA* plasmid [35]. The plasmid was transformed into the *E. coli* DH10B strain (Thermo Fisher Scientific Baltics). The resulting bacteria were grown in LB medium with 100 µg/mL of ampicillin at 37 °C until OD_600_ reached around 0.8; the IPTG inducer was added to the final concentration of 0.5 mM, and the culture was grown for another 3 h. Cells were collected by centrifugation and resuspended in 20 mM Tris-HCl, pH 7.9, with 0.3 M NaCl. Then cells were disrupted by 5 min. sonication at 22 kHz in 10 s periods with 10 s cooling intervals, using 40% amplitude. Cell debris was removed by centrifugation at 12,000× *g* for 30 min. The resulting supernatant was loaded onto an Ni-NTA column (Cytiva Sweden AB, Uppsala, Sweden), previously equilibrated with 20 mM Tris-HCl, pH 7.9, with 0.3 M NaCl. Proteins were applied to the column and washed with 2 column volumes of the equilibration buffer. The absorbed proteins were eluted with 20 mM Tris-HCl, pH 7.9, with 0.3 M NaCl using a linear gradient of 0–250 mM of imidazole. Protein-containing fractions were pooled and desalted against 20 mM Tris-HCl, pH 7.9. Purity of recombinant CodA was confirmed by SDS-PAGE electrophoresis (Figure S6). The concentration of protein was measured using the Bradford method.

The enzymatic reactions were carried out at 25 °C in 20 mM Tris-HCl, pH 7.9, for 30 min. A 30 µL final reaction mixture contained 1 µg (leading to the final concentration of around 0.6 µM) of purified protein and 20 mM substrate. After incubation, 1 µL of the reaction mixture was used for thin-layer chromatography analysis that was performed on aluminum sheets coated with silica gel 60 F_254_ using a chloroform/methanol mixture (5:1) as a mobile phase. The spots were visualized under 254 nm UV light.

## 4. Conclusions

We demonstrated in this study that *N*^4^-methylcytosine, but not *N*^4^,*N*^4^-dimethylcytosine, supports the growth of the *E. coli* ∆*pyrF*::Km auxotroph in minimal M9 media. Further experiments demonstrated that the generation time of this strain in M9 medium supplemented with *N*^4^-methylcytosine is approximately 3 h compared to approximately 1.5 h for cytosine or uracil. In contrast, little or no growth was observed for *N*^4^,*N*^4^-dimethylcytosine, with a calculated generation time of at least 40 h. We found that around 12% of *N*^4^-methylcytosine is consumed and presumably converted into cytosine and further into uracil after 24 h of growth in M9 medium. Purification of the CodA deaminase and determination of its enzymatic activity demonstrated that, in contrast to cytosine, neither *N*^4^-methylcytosine nor *N*^4^,*N*^4^-dimethylcytosine is converted into uracil. Therefore, we propose that *N*^4^-methylcytosine is not directly converted into uracil by CodA to support the growth of *E. coli*, but instead it is converted first into cytosine by yet unknown enzyme(s). The enzymatic pathway for such conversion is proposed. Further experiments to determine the gene encoding the N-demethylase enzyme are described.

## Figures and Tables

**Table 1:** Generation time of the *E. coli* ∆*pyrF*::Km strain in liquid M9 media with different heterocyclic bases. Values represent the average of 3 experiments and the standard deviation.

	Uracil	Cytosine	*N*^4^-MethylC	*N*^4^,*N*^4^-DimethylC
Generation time, h	1.53 ± 0.04	1.49 ± 0.10	2.99 ± 0.59	33.03 ± 11.41

## Data Availability

The original contributions presented in this study are included in the article/Supplementary Material; further inquiries can be directed to the corresponding author.

## Supplementary Materials

The following supporting information can be downloaded at: https://www.mdpi.com/article/10.3390/ijms26051812/s1.
