# Supplementary material for: N4-Methylcytosine Supports the Growth of Escherichia coli Uracil Auxotrophs
Source: Int J Mol Sci. 2025 Feb 20;26(5):1812. doi: 10.3390/ijms26051812 (PMC11898903; doi:10.3390/ijms26051812)
Supplement: Supplementary file 1 [file ijms-26-01812-s001.zip › ijms-3478161-supplementary.pdf]

SUPPORTING INFORMATION

N^4-methylcytosine supports the growth of *Escherichia coli* uracil auxotrophs

Jaunius Urbonavičius [1], Aušrinė Čekytė [1], and Daiva Tauraitė [1*]

Department of Chemistry and Bioengineering, Vilnius Gediminas Technical University, Saulėtekio Av. 11, 10223 Vilnius, Lithuania; jaunius.urbonavicius@vilniustech.lt; ausrinecekyte@gmail.com

*Correspondence: daiva.tauraite@vilniustech.lt; Tel.: +37052744839

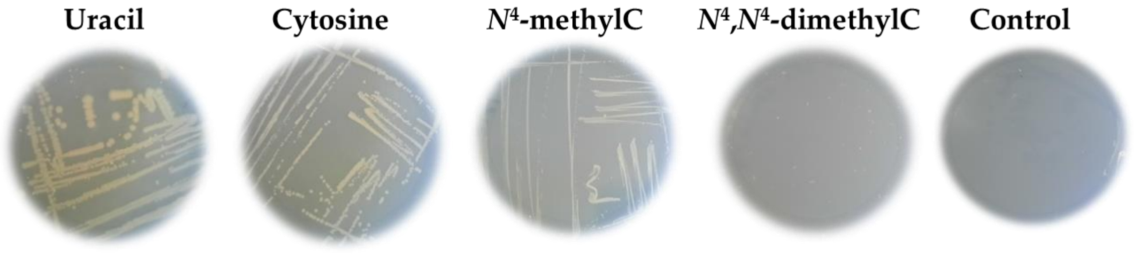

Figure S1. Growth of *S. typhi ΔpyrF::*Tn10 strain on the M9 agar minimal medium supplemented with either uracil, cytosine, N^4-methylcytosine, N^4,N^4-dimethylcytosine, or without any heterocyclic base (control). The agar plates were incubated at 37 °C for 72 hours.

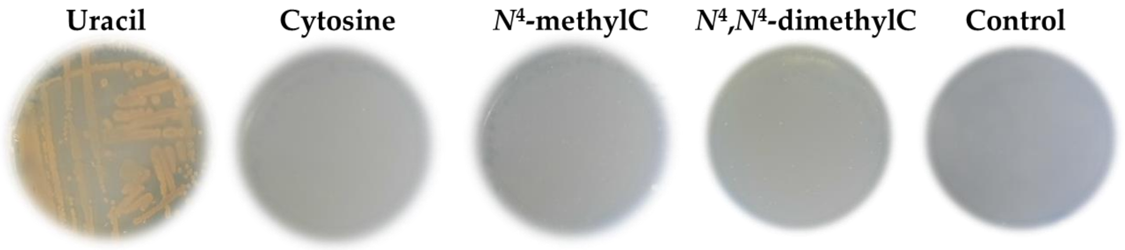

Figure S2. Growth of *B. subtilis ΔpyrF*::erm strain on the M9 agar minimal medium supplemented with either uracil, cytosine, N^4-methylcytosine, N^4,N^4-dimethylcytosine, or without any heterocyclic base (control). The agar plates were incubated at 37 °C for 72 hours.

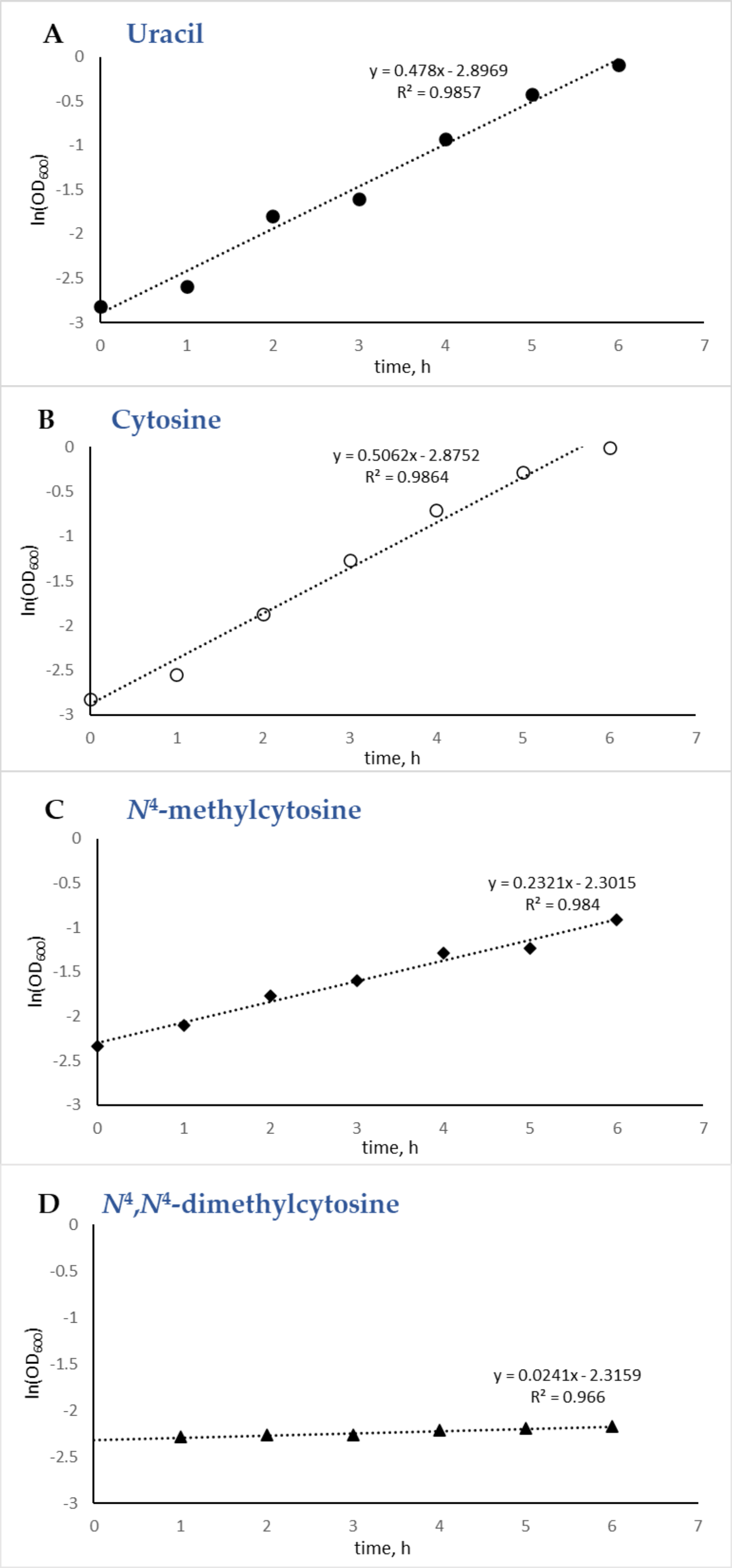

Figure S3. Semi-logarithmic plot of growth of *E. coli ΔpyrF*::Km strain in the M9 liquid medium supplemented with: **A**- uracil; **B**- cytosine; **C**- *N*⁴-methylcytosine; **D**- *N*⁴,*N*⁴-dimethylcytosine.

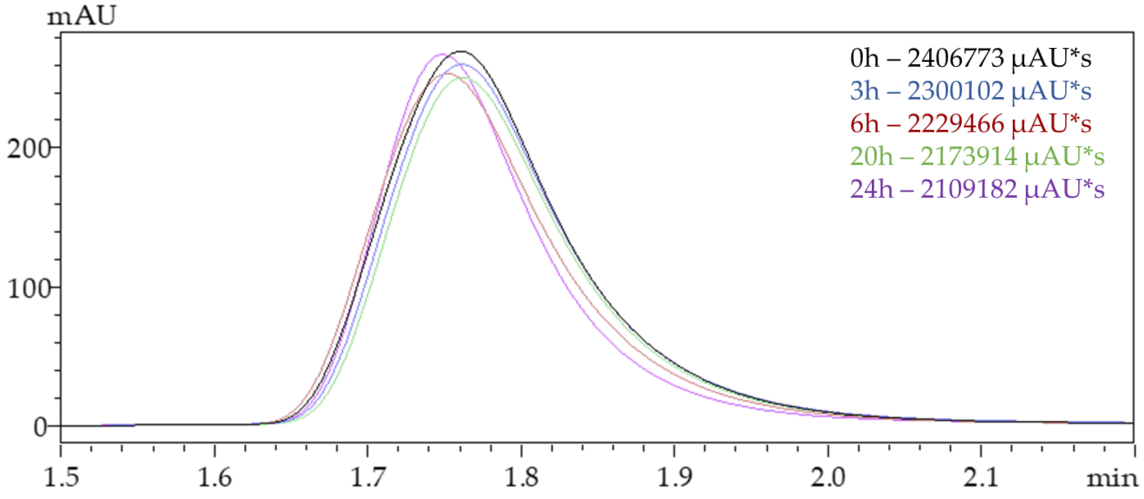

Figure S4. HPLC chromatograms of N^4-methylcytosine peak area that decreases during the first 24 h of growth of *E. coli ΔpyrF*::Km strain in M9 liquid medium. The value of peak area (μAU *s) are inserted on the right.

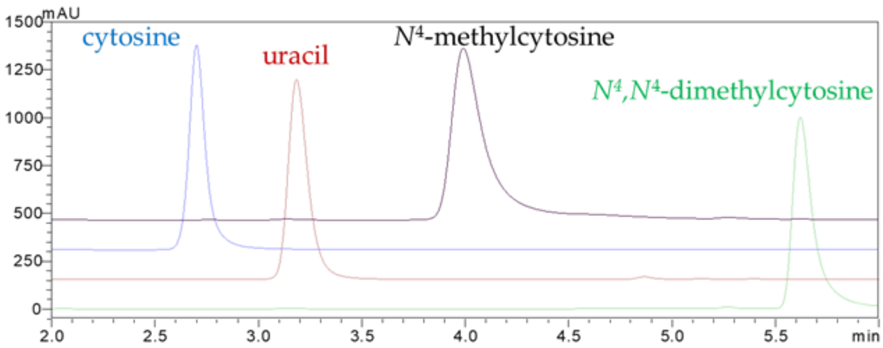

Figure S5. HPLC chromatograms showing the stability of N^4-methylcytosine and N^4,N^4-dimethylcytosine after 72 incubation in the M9 liquid medium without *E. coli ΔpyrF*::Km strain. Chromatograms of cytosine and uracil are inserted as reference.

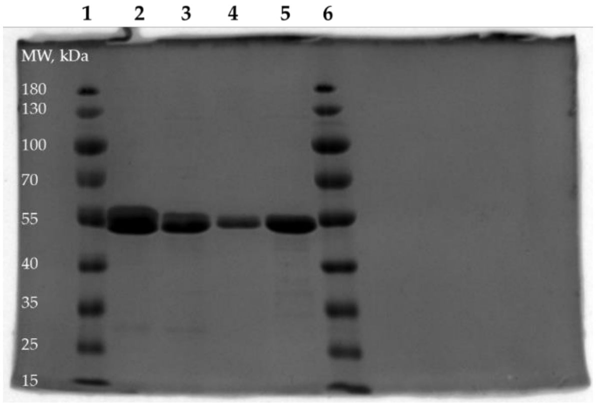

Figure S6. SDS-PAGE analysis of purified recombinant CodA. Twelve % acrylamide gel was used and stained with Coomassie blue. Lanes 1 and 6 – molecular weight marker. Lanes 2-5 – fractions eluted with 100-150 mM imidazole in 20 mM Tris-HCl, 0.3 M NaCl, pH 7.9 buffer.
